# Color doppler ultrasound for the assessment of palatal fibromucosa thickness and the trajectory of the greater palatine artery: A pilot study

**DOI:** 10.4317/jced.59704

**Published:** 2022-07-01

**Authors:** Raúl Sampietro-Martínez, Javier Pérez-Monreal, Alba Sánchez-Torres, Javier Bara-Casaus, Cosme Gay-Escoda

**Affiliations:** 1 DDS. MS. Master Degree Program in Oral Surgery and Orofacial Implantology (EFHRE International University/FUCSO); 2 MD, MSc, PhD, EBPh. Director of the Department of Vascular Doppler Ultrasound, MAZ Hospital. Zaragoza, Spain. Associate Professor of the Master Degree Program in Phlebology and Lymphology, University of Alcala. Madrid, Spain; 3 DDS, MS. Master of Oral Surgery and Orofacial Implantology. Associate Professor of the Oral Surgery Department, School of Dentistry, University of Barcelona, Spain; 4 MD, PhD, OMFS. Director of the Maxillofacial Institute of Sagrat Cor University Hospital, Barcelona, Spain. Co-Director of the Specialist Course in TMJ and Orofacial Pain, University of Barcelona. Director of the Postgraduate Course in Oral Medicine and Surgery of the Catalan Society of Odontology and Stomatology; 5 MD, DDS, MS, PhD, EBOS, OMFS. Chairman and Professor of the Oral and Maxillofacial Surgery Department, School of Dentistry, Uni¬versity of Barcelona. Director of the Master Degree Program in Oral Surgery and Implantology (EFHRE International University / FUCSO). Coordinator/Researcher of the IDIBELL Institute. Head of the Oral and Maxillofacial Surgery and Implantology Department, Teknon Medical Center, Barcelona, Spain

## Abstract

**Background:**

The primary objective of this study was to determine the position and course of the greater palatine artery using color doppler ultrasound. The secondary objective was to determine the thickness of the palatine fibromucosa.

**Material and Methods:**

A pilot case series study was performed in a private clinic during February 2020. The scans were performed with a Mindray® M9 ultrasound machine (Mindray North America, NJ, USA) coupled to an L16-4Hs® hockey-type angled probe. For each participant, the arterial path and thickness of the palatal fibromucosa were determined at 5 different points.

**Results:**

A total of 6 volunteers (3 males and 3 females) with a mean age of 39.2 (±16) years were included. While the thickness of the fibromucosa decreased along the anterior area, the distance from the cementoenamel junction to the position of the artery was generally maintained up to the canine position, where it was found to be closed to teeth.

**Conclusions:**

Color doppler ultrasound allows accurate localization of the artery as well as measurement of the thickness of the palatine fibromucosa. It would help to select the best area for graft harvesting in order to avoid bleeding complications due to vascular sectioning.

** Key words:**Hard palate, doppler ultrasonography, diagnosis, connective tissue graft.

## Introduction

Soft tissue autografts through the addition of keratinized gingiva and/or connective tissue are widely used in periodontics and oral surgery both for the treatment of gingival recessions and for gaining volume in oral rehabilitations by means of osseointegrated implants. The palate is the most frequent donor site for the procurement of these autografts, either the free gingival graft - a surgical technique described by Sullivan and Atkins (1) and later modified by Miller (2), or the subepithelial connective tissue graft, described by Langer and Langer (3).

Before harvesting the autograft, it is advisable to palpate the palatal bony sulcus containing the neurovascular bundle, with the aim of establishing an apical limit of the incision in order to avoid sectioning this anatomical structure. The neurovascular bundle containing the greater palatine artery (GPA) emerges through the greater palatine foramen at the level of the second and third molars, approximately at the midpoint between the bony crest and the palatal raphe, and running after its emergence in an anterior direction. It is also convenient to measure the thickness of the palatal fibromucosa in order to know the volume of tissue available to cover the receptor area. In this sense, Miller (2) recommended a minimum thickness of the palate of 4 mm for connective tissue grafts. Regarding the volume of the graft, Allen (4) concluded that a considerable volume of graft (from 1.5 mm in thickness) seems to produce a higher survival rate.

Reiser (5) described some measures of GPA location in relation to the cementoenamel junction (CEJ). When the donor area is located in a low or flat palatal vault, the artery is normally located in a position closer to the CEJ (about 7 mm). In mid-palates it is located about 12 mm from the CEJ, and in case of U-shaped or ogival palates the distance is approximately 17 mm. In another study by Yu *et al*. (6), the most appropriate donor site for the procurement of gingival autografts was found to be the region between 3-9 mm below the CEJ, between the distal surface of the canine and the midline surface of the first molar. However, the localization of the GPA by these measurements does not always yield the expected result, and sometimes the GPA is sectioned and may cause profuse bleeding during and after surgery.

There are pilot studies (7,8) that measure the thickness of the palatine fibromucosa and the location of the palatine artery by means of magnetic resonance imaging (MRI). Hilgenfeld *et al*. (7) evaluated whether high-resolution, non-contrast dental MRI could be used for the accurate determination of palatal fibromucosa thickness and for localization of the GPA. They observed that the thickness of the palatal fibromucosa measured by MRI was comparable to that obtained by bone probing. These authors therefore concluded that dental MRI allows reliable, noninvasive, radiation-free planning to preoperatively establish the thickness of the fibromucosa and the location of the GPA at 85% of the measurement sites - which could reduce intraoperative complications.

Noninvasive and radiation-free observation of oral tissues is also possible through ultrasound techniques. There are several types of ultrasound devices: A-mode (one-dimensional mapping of the interfaces between tissues using echo techniques), B-mode (two-dimensional gray-scale imaging of the scanned area using echo techniques), M-mode (used for moving structures, obtaining a graphic representation of the signal, where amplitude is the vertical axis, and time and depth are the horizontal axis), doppler mode (characterized by frequency modification in a transmitted wave detected by a receiver due to certain mobility conditions between the receiver, the transmitter, the medium and the reflecting tissues), and finally color doppler mode. In this mode the transducer is energized by a relatively short pulse and the doppler information obtained along the line of sight of the transducer is displayed in color instead of a gray scale as used in B-mode imaging (9,10)

The pioneers in the use of ultrasound as a diagnostic tool in dentistry were Braum *et al*. (11) They obtained images through an ophthalmic ultrasound device (15 MHz) of the internal structures of the teeth. In 1967, Smirnow *et al*. (12) and Reich *et al*. (13) used pulse-echo methods for the study of the oral cavity. Kossoff *et al*. (14) used direct transmission ultrasound to study the properties of the pulp cavity. Later, Daly *et al*. (15) conducted research on the application of ultrasound to explore soft tissues in dentistry. Kydd *et al*. (16) succeeded in measuring gingival thickness by ultrasound and Spranger *et al*. (17) used ultrasound for the first time for the diagnosis of periodontal disease. Palou *et al*. (18) used an ophthalmic ultrasound device to evaluate periodontal bone morphology. They chose this device because it involved the smallest probe available (6 mm) - but the fact that the ophthalmic probe was a straight bar probe made it difficult to use in areas distal to the premolar and lingually, and led the authors to postulate that using an angled probe similar to a dental contra-angle handpiece might be more appropriate for the oral cavity. Another study by Eger *et al*. (19), in which the gingival biotype was measured using ultrasound, found the diagnostic performance of this test to be excellent.

Several authors have measured the palatal fibromucosa using ultrasound devices with sufficient accuracy to measure its thickness quickly and noninvasively. Among them, Müller *et al*. (20,21) and Rajpoot *et al*. (22) performed measurements using the echo-pulse principle; Schulze *et al*. (23) compared the accuracy of mode A and mode B ultrasound with manual probing for the measurement of palatal fibromucosa thickness, obtaining similar results; and Salmon *et al*. (24) performed a pilot study to test the performance of a 25 MHz high-frequency ultrasound probe to measure the biological width - currently known as supracrestal attached tissue.

Surgery to obtain autografts from the palatal area requires previous exploration to know the amount of tissue we can have from the donor area and avoid intraoperative and postoperative complications. Sectioning of the GPA is a potential complication in surgery to obtain palatal autografts; its localization by color doppler ultrasound and mapping prior to surgery therefore could be essential to avoid damage to this anatomical structure.

The primary objective of this study was to determine the exact position and trajectory of the GPA using color doppler ultrasound. The secondary objective was to determine the thickness of the palatine fibromucosa.

## Material and Methods

This manuscript was written in accordance with the Strengthening the Reporting of Observational studies in Epidemiology (STROBE) statement (25). The present work is a pilot case series study from a private clinic conducted during the month of February 2020. The study protocol was approved by the Clinical Research Ethics Committee of Aragón (CEICA) (Spain) with minute number 02/2020. All patients signed an informed consent for participation in the study and performance of the ultrasound examinations. To be included, patients had to be of legal age, and the exclusion criteria were the presence of any oral or systemic disorder with repercussions upon the palatal fibromucosa.

Intraoral ultrasound scans to determine the course of the GPA as well as the thickness of the palatine fibromucosa were performed with a Mindray® M9 ultrasound scanner (Mindray North America, NJ, USA) coupled to an L16-4Hs ® hockey-type angled ultrasound probe for musculoskeletal, superficial, neurological and vascular applications. The exact characteristics of the probe can be seen in Table 1. The scans were performed by a specialist in vascular ultrasound (JPM) who collaborated in carrying out the study. The scans were performed using a frequency of 13.5 MHz in B-mode and 6.6 MHz in color doppler mode. Hyaluronic acid gel was used for correct displacement and image capture by the ultrasound probe.

**Table 1:**
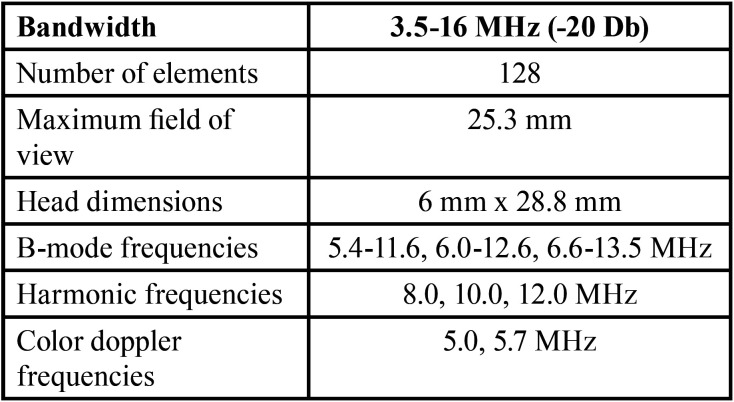
Physical characteristics of the ultrasound probe.

**Figure 1:**
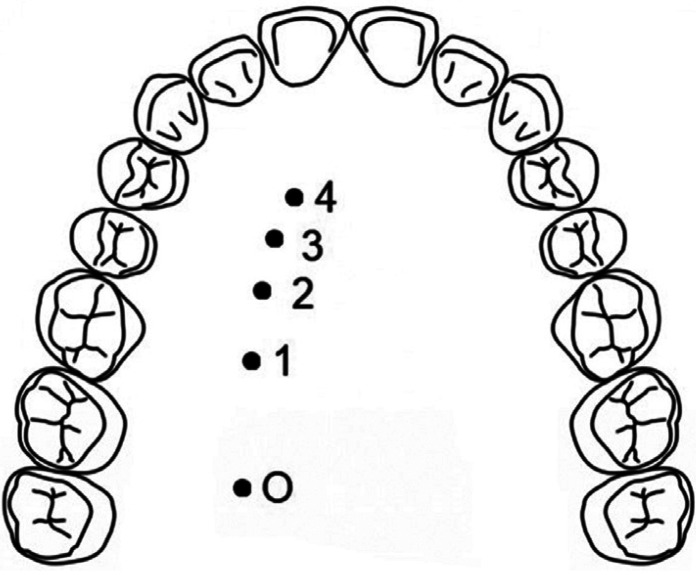
Reference points for recording of the arterial path and palatine fibromucosa thickness: origin of the greater palatine artery (O), midpoint of the first molar (1), midpoint of the second premolar (2), midpoint of the first premolar (3), midpoint of the canine (4).

**Figure 2:**
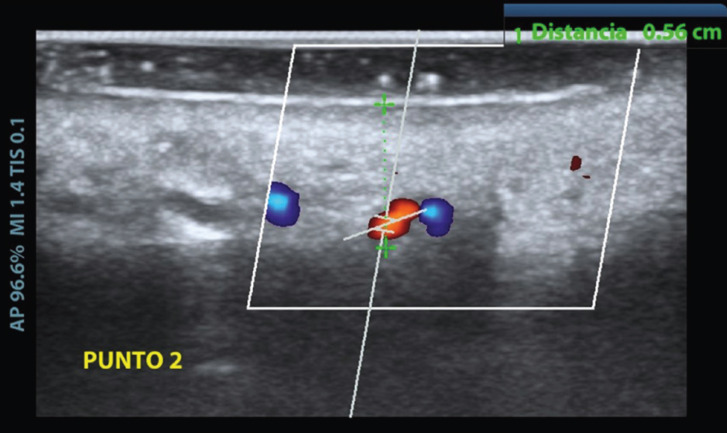
Measurement of the thickness of the palatine fibromucosa (distance from the epithelium to the palatine cortex).

To determine the path of the GPA, 5 reference points were established as shown in Figure 1: origin of the GPA (O), midpoint of the first molar 1), midpoint of the second premolar 2), midpoint of the first premolar 3), and midpoint of the canine 4). The ultrasound probe was used to locate the emergence of the neurovascular bundle. At each of the reference points, the presence of pulsatile blood flow (velocimetry curve) and the doppler effect were determined. Once these two features were located, the image of the arterial segment was centered and made to correspond with the center of the probe, thus ensuring that the tissue underlying the probe contained the GPA, and the image provided by the ultrasound scanner was recorded. Using the ultrasound measuring system, the thickness of the fibromucosa (distance from the epithelium to the palatal cortex) was measured at each of the preset points (Fig. 2), and the path of the GPA (distance from the CEJ to the reference point) was marked with a Tondaus® gentian violet surgical marker (Kapok Stationery Co., Guangdong, China). In order not to lose the reference of the arterial position, the tip of a periodontal probe was rested just at the point where the artery had been located with the ultrasound probe. This maneuver was performed without lifting the ultrasound probe completely so as not to lose the reference of the exact point where the artery was located.

## Results

A total of 6 volunteers (3 men and 3 women) with a mean age of 39.2 (±16) years were included in the study, obtaining a total of 30 ultrasound images (5 images per participant) of the preset points.

**Figure 3:**
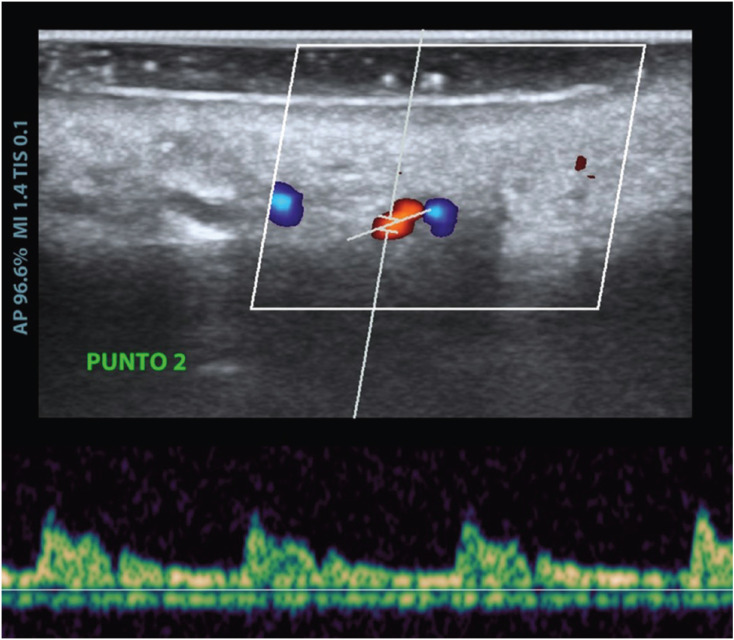
Velocimetry curve and doppler effect (red and blue).

Firstly, the emergence of the GPA was located. Next, the velocimetry curve and the doppler effect were determined at each of the reference points, as shown in Figure 3. As the exact area where the artery was located was recorded, the path was marked with the surgical marker.

Table 2 reports the thickness of the palatal fibromucosa at the different points for each participant. The results show a progressive decrease in thickness from point O to the most anterior area.

**Table 2:**
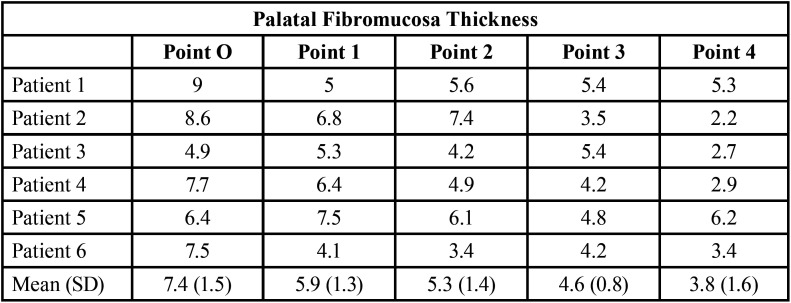
Palatal fibromucosa thickness measurements (in mm) for each of the patients included.

Table 3 shows the path of the GPA measured as the distance from the CEJ to the artery at each of the preset points. It only shows a slight decrease in distance from the most posterior to the most anterior positions. In fact, the distance was overall maintained up to point 4, i.e., at canine level, where the GPA lies closer to the teeth.

**Table 3:**
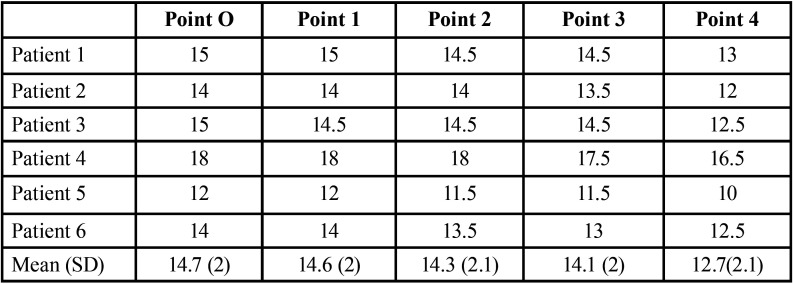
Greater palatine artery path measured as the distance from the cementoenamel junction to the artery.

**Figure 4:**
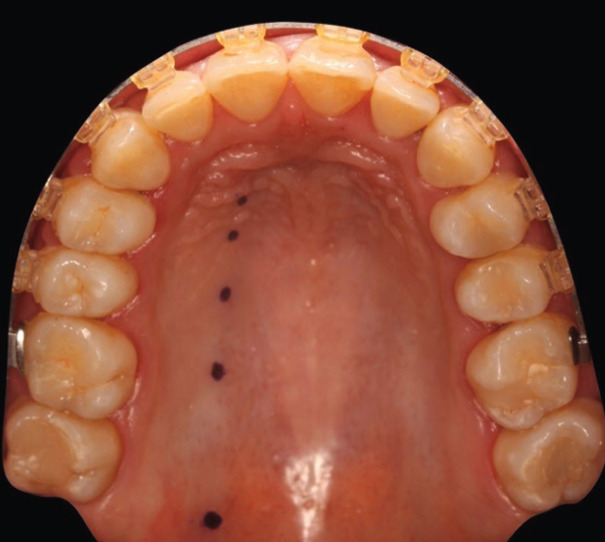
Trajectory of the greater palatine artery marked with a surgical marker pen.

Once we had located and marked the 5 points corresponding to the course of the GPA with the surgical marker, we took an intraoral photograph of the course of the artery in each of the participants (Fig. 4).

## Discussion

The thickness of the palatine fibromucosa had been previously explored using B-mode ultrasound techniques (23,24). However, to our knowledge, this is the first study using color doppler ultrasound to locate and map the course of the GPA. The study by Schulze *et al*. (23) explored the thickness of the fibromucosa by ultrasound at a single point per patient at the level of the molars. Other studies on masticatory mucosal thicknesses (26,27) have scanned a larger number of points per patient. These studies scanned the mucosa of the canine, first premolar, second premolar, first molar and second premolar at 3, 6, 9 and 12 mm from the CEJ. However, they were performed using 3D radiology and not ultrasonography, since it is technically easier to measure more points through radiographic means than through ultrasound. In our study we evaluated the thickness of the fibromucosa at the sites where the artery was located in each of the preset areas (first molar, second premolar, first molar and canine). During the ultrasound measurements, we encountered a series of problems or difficulties regarding handling of the ultrasound probe in the oral cavity. It should be remembered that the probe, although with an angled shape as proposed in the study by Palou *et al*. (18), and being one of the smallest ultrasound probes with the capacity to visualize very superficial tissues, is not specifically designed for intraoral use, since it was originally intended for dermatological, neurological, musculoskeletal and vascular use, and therefore with the capacity to work in doppler type frequencies. There are specific ultrasound probes for intraoral use (24,28). However, these probes have not been used in doppler type frequencies in the oral cavity. The handling of the probe used in the present study is especially difficult in patients with an ogival palate, especially in the anterior part, which can result in distorted images of the anatomical limits (epithelium and palatal cortex) and make it difficult to fix a precise limit in the images of the anterior part of the palate. In this sense, it would be interesting to carry out studies similar to our own with specific ultrasound probes for intraoral use in order to determine whether probes of this kind make exploration of the anterior part of the palate easier for the operator. Likewise, in order to guarantee maximum precision regarding the thickness of the fibromucosa, it would be necessary to compare the results with another type of tool such as measurement of the fibromucosa with a periodontal probe. In this sense, Schulze *et al*. (23) already concluded that ultrasound is a method as reliable in the exploration of the fibromucosa as manual probing. The results obtained in our study, which although showing a slight progressive decrease between the CEJ and the GPA, the measurements remain constant as the palate advances (until reaching the level of the canines) are in agreement with other studies (5,29) that evaluate the trajectory of the GPA. Likewise, the doppler image and pulsatile flow can ensure its position, though it depends largely on the ability of the operator to mark it by means of a periodontal probe and surgical marker. The clinical uses which dentists can obtain from scanning prior to graft surgery are manifold. The present ultrasound technique allows a better therapeutic decision to be made regarding the use of autografts compared to other alternatives. In addition, it allows us to determine the best area for graft harvesting in order to avoid bleeding complications due to sectioning of the GPA. Of course, handling the ultrasound scanner requires a learning curve to achieve reliable results. In this sense, dentists should be specifically trained in this technique or have a specialist in this field of medicine to ensure correct exploratory results.

## Conclusions

The results of this pilot study indicate that accurate localization of the GPA is possible by means of color doppler ultrasound, as well as the measurement of the thickness of the palatine fibromucosa. The technique allows a better therapeutic decision to be made regarding the use of autografts compared to other alternatives. In addition, it is able to define the best area for graft harvesting in order to avoid bleeding complications due to sectioning of the greater palatine artery.
